# Methanol fixed fibroblasts serve as feeder cells to maintain stem cells in the pluripotent state in vitro

**DOI:** 10.1038/s41598-018-26238-2

**Published:** 2018-05-17

**Authors:** Yahui Ren, Ziyu Ma, Tong Yu, Min Ling, Huayan Wang

**Affiliations:** 1 Department of Animal Biotechnology, College of Veterinary Medicine, Northwest A&F University, Yangling, Shaanxi 712100 China; 2 Department of Innovation Experimental College, Northwest A&F University, Yangling, Shaanxi 712100 China

## Abstract

Preparation of mouse embryonic fibroblast (MEF) feeder cells to maintain pluripotent stem cells (PSCs) is time consuming and involved in animal issues. Here, we demonstrated a novel method to prepare feeder cells with high efficiency, timesaving, and low costs. MEFs in 3 × 10^4^ cell/cm^2^ were fixed by methanol for 5 min and air drying for 5 min. Thereafter, the methanol fixed MEF cells (MT-MEF) were able to be used directly to culture PSCs or stored at room temperature for the future usage. PSCs cultured on MT-MEF could be continuously expanded for over 40 passages with the naïve pluripotency. MT-MEFs could also be used to maintain human and pig iPSCs. Moreover, methanol fixed MEFs’ culture dish was able to be reused for at least 4 times, and to be applied for antibiotic resistant screening assay to establishing stable transfected PSC lines. Alternatively, the immortalized cell lines, for instance NIH3T3 cells, could also be fixed by methanol and used as feeder cells to maintain PSCs. Thus, this novel means of methanol fixed feeder cells can completely replace the mitomycin C and gamma radiation treated MEF feeder cells, and be used to maintain PSCs derived from mouse as well as other animal species.

## Introduction

Pluripotent stem cells (PSCs), including embryonic stem cells (ESCs) and induced pluripotent stem cells (iPSCs), have a great promise in regenerative medicine, disease modeling, and cell therapies^1–3^. To culture PSCs, either mitomycin C (MMC) or gamma radiation treated mouse embryonic fibroblasts (MEFs) were commonly used as feeder cells to maintain the self-renewal and pluripotency^4–6^. Recently, expanded/extended potential stem cells (EPSCs) that contribute to both embryo proper and placenta trophoblasts in chimeras, were also established and cultured on MEF feeder cells^7,8^. The speculated reasons of using MMC-MEFs were due to that MEFs might produce and secrete growth factors, including leukemia inhibitory factor (LIF), fibroblast growth factor (FGF), and bone morphogenetic protein (BMP) etc.^9–11^, to maintain PSCs in the naïve pluripotent state. However, there were many inadequacies of using MMC and radiation treatment of MEF feeder cells. First, the preparation of MEFs is a complex and time consuming process^12,13^. Second, the MMC is pricey and residual MMC might produce cytotoxicologial effects on ESCs^14^. Additionally, application of gamma radiation requires the special equipment and devices^15^. Third, animal-derived MEFs retain the xenogeneic components that limit its application to culture human PSCs that may use to treat debilitating human diseases^16,17^. Therefore, feeder-free culture systems are the alternative approaches to replace MEF feeder cells. Culture dishes coated with the recombinant and synthesized macromolecules, including gelatin^18^, Matrigel^19^, recombinant extracellular matrix proteins^20–22^, synthetic polymers^23,24^, hydrogel^25,26^, recombinant E-cadherin substratum^27^, Glycosaminoglycan^27^, and Oligopeptide^28^, as well as 3D scaffold^28–30^, were developed and used to culture PSCs. However, these methods either use animal products that may have potential problems in transplantation applications or need special growth factors and media.

Recently, reports showed that chemicals glutaraldehyde (GA) and formaldehyde (FA) were able to fix feeder cells that were used to maintain the pluripotency of mouse and human PSCs^31–33^. The procedures of chemical fixation with GA and FA required to wash out GA and FA residues by PBS for multiple times, and then the fixed cells could be stored at 4 °C or freeze-dried first and stored at room temperature for further usage^31–33^. The principle concept of GA and FA fixation of feeder cells may provide a convenient method to replace the traditional method to make feeder cells.

Extracellular matrix (ECM) influences adhesion, migration, differentiation and proliferation of stem cells through communicating with cell surface receptors and adhesion molecule such as integrins^34–36^. Methanol-fixed feeder cells, which are unable to produce growth factors and cytokines that PSCs required, still retain ECM proteins in the surface of fixed cells and provide niches and signaling for PSCs to control the balance between self-renewal and differentiation. Collagenase-IV is one of the matrix metalloprotinase, which degrades ECM proteins such as collagen-IV, fibronectin, laminin, and vitronectin^37^. Thus, the treatment of collagenase-IV is able to remove collagen-IV and fibronectin from the surface of methanol fixed feeder cells. Consequently, the pluripotency and adhesion ability of PSCs may be affected when cells are cultured on the collagenase-IV treated methanol fixed feeder cells.

In this study, we develop a novel method to maintain PSC self-renewal and pluripotency for the long-term expansion. Methanol-fixed feeder cells not only were used to culture mouse, human, and porcine pluripotent stem cells, but also were used for antibiotic-resistant screening and repeated usage. Meanwhile, we demonstrated that ECM proteins collagen-IV and fibronectin were crucial for PSCs attachment and maintaining naïve state pluripotency of PSCs.

## Results

### Culture of mouse ES on methanol-fixed feeder cells

**Figure 1:**
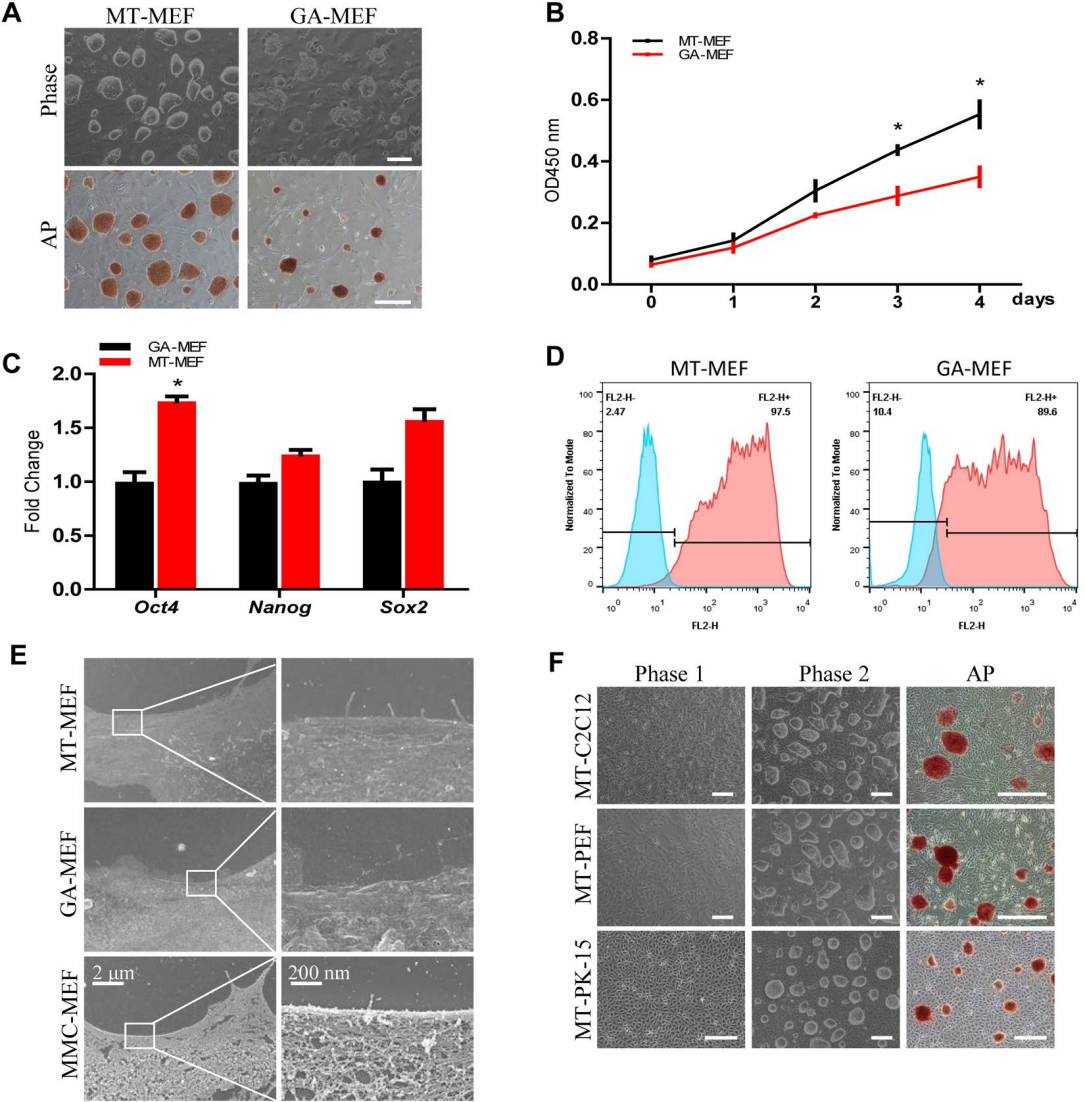
Culture of mouse ES on fibroblasts fixed by methanol. J1 mES cells were cultured on MEF cells fixed by MT (MT-MEF) and GA (GA-MEF), respectively. (**A**) Morphology and AP staining of J1 mES cells. (**B**) Growth curve of J1 cells. (**C**) qRT-PCR analysis of *Oct4*, *Nanog*, and *Sox2* expressions in J1 cells. (**D**) Flow cytometry analysis of SSEA-1 expression in J1 cells. (**E**) Scanning electron microscope analysis of MT-MEF, GA-MEF, and MMC-MEF. (**F**) J1 cells were cultured on MT fixed C2C12, PEF, and PK-15 cells. Phase 1, MT fixed cells; Phase 2, morphology of J1 cells cultured on MT fixed cells. Scale bar, 200 μm. Data indicate mean ± SD, *P < 0.05, n = 3.

The previous reports showed that cells fixed by glutaraldehyde (GA) and paraformaldehyde could be used as feeder cells to maintain mouse and human induced pluripotent stem cells^31–33^. To verify the chemical fixation method, we prepared feeder cells by GA following the procedure reported by Yue, X. S.^32^. The results showed that GA procedure was time consuming, and results were jagged. Alternatively, we used 100% methanol to fix cells, which is the first time reported, and found that methanol fixed MEF (MT-MEF) cells were able to maintain mouse PSCs. The J1 mES cells cultured on MT-MEF presented more domed and compact morphology compared to the control GA fixed MEF (GA-MEF) (Fig. 1A). Growth rate of J1 mES on MT-MEF was significantly increased versus on control GA-MEF (Fig. 1B). The expression level of *Oct4*, *Nanog*, and *Sox2* was increased in J1 cells when cultured on MT-MEF (Fig. 1C). The ratio of SSEA-1 positive cell population could reach 97.5% on MT-MEF versus 89.5% on GA-MEF feeders (Fig. 1D). Additionally, the scanning electron microscope analysis of MT-MEF, GA-MEF, and MMC-MEF showed that the cellular membrane of MT and GA fixed cells presented the smooth surface, but the MMC treated cell membrane displayed the net-like structure, indicating that chemical components indeed changed the cellular membrane structure of fixed cells (Fig. 1E), in which the cytoplasmic proteins may be released to the surface of cell membrane and play a role for PSC adhesion.

Besides MEF, we also used epithelial cell PK-15, myoblast C2C12, and porcine embryonic fibroblast (PEF) fixed by methanol to culture J1 mES. Results showed that J1 mES could be maintained naïve morphology and alkaline phosphatase (AP) activity on MT fixed cells no matter cell types and where cells coming from (Fig. 1F). As control experiments, we did the parallel assays by culturing J1 cells on plates coated with gelatin, Matrigel, laminin, and poly-L-Lysine (Fig. S1). Results indicated that methanol fixation method is easier to handle, low cost, and shows the better result of maintenance of PSC pluripotency.

### Optimizing the methanol-fixed method

**Figure 2:**
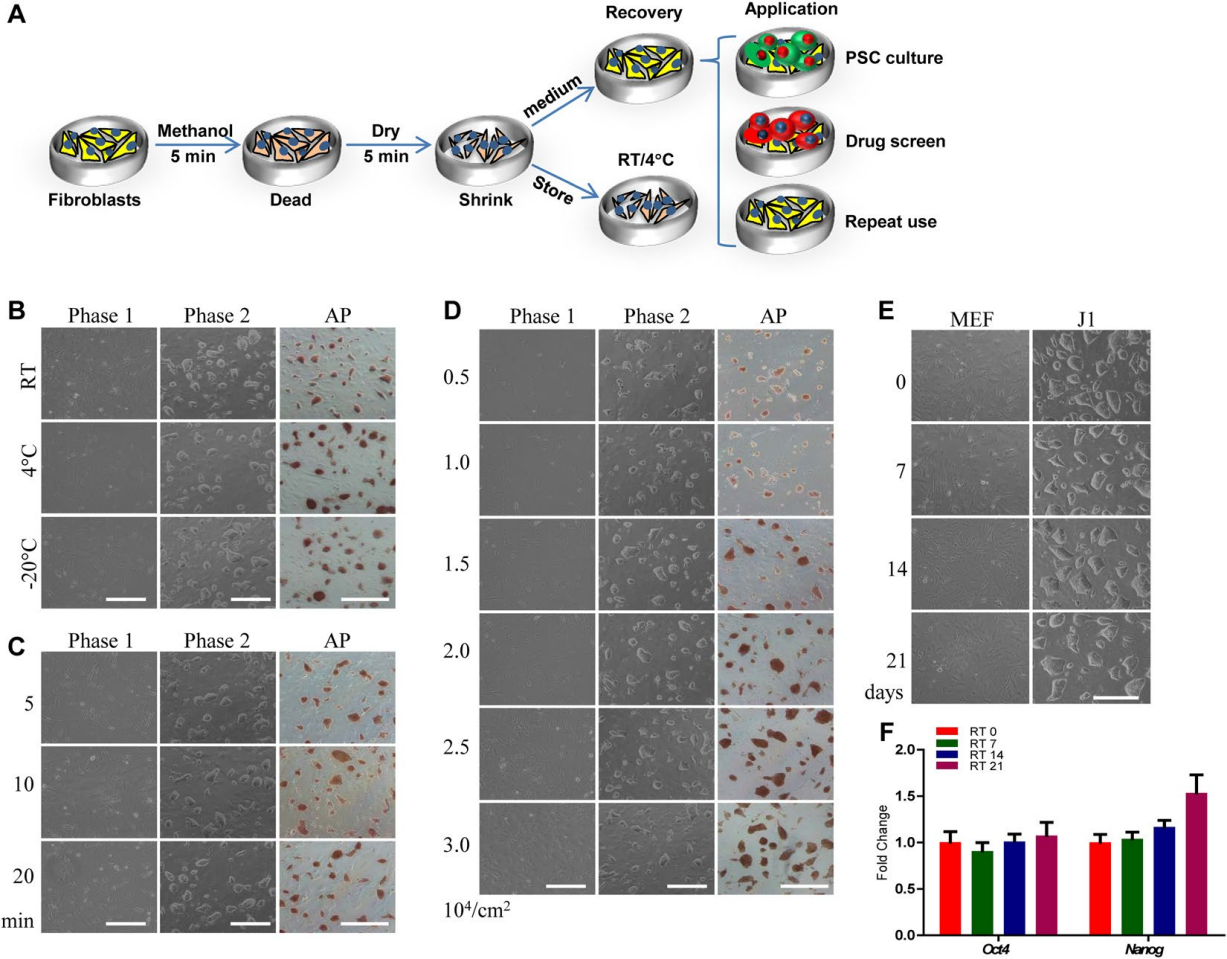
Optimizing procedure of methanol fixation. The J1 mES cells were cultured on methanol fixed MEF cells (MT-MEF) under different conditions. (**A**) Schematic diagram of procedure and application of MT-MEF. (**B**) Methanol in different temperatures was used to fix MEFs. (**C**) MEFs were fixed by methanol for 5 to 20 min. (**D**) MEFs in the density from 5 × 10^3^/cm^2^ to 3 × 10^4^/cm^2^ were fixed by methanol. (**E**) MT-MEFs stored at room temperature (RT) for 0 to 21 days. (**F**) qRT-PCR analysis of *Oct4* and *Nanog* expressions in J1 cells cultured on MT-MEF that was stored at RT for different dates (0 to 21 days). Phase 1, morphology of MT-MEF; Phase 2, morphology of J1 cells cultured on MT-MEF. Scale bar, 400 μm for B–D, 200 μm for E.

Because of the application of methanol and acetone in histochemistry and cytochemistry, we initially mixed methanol and acetone in the ratio of 5:5 and used to fix MEF cells. The J1 cells could be maintained on methanol and acetone (5:5) fixed MEF cells for long term passages with the domed morphology and AP positive staining (Fig. S2A). We then used the different proportion of methanol and acetone to fix MEF cells and detected the effect on the culture of pluripotent stem cells. Results of cell morphology and AP staining showed that when MEF cells were fixed by methanol and acetone at ration of 3:7, the AP staining of J1 cells was faded (Fig. S2A), the *Oct4* and *Nanog* expressions were decreased (Fig. S2B), and the number of AP positive colonies was reduced (Fig. S2C). Alternatively, when use of methanol only to fix MEF cells, J1 cells were maintained excellently (Fig. S2A). Based on this observation, acetone was removed from formula and methanol solely was used to fix feeder cells. To test the efficacy of methanol concentration, MEF and NIH3T3 cells were treated with different concentration of methanol from 10% to 100%. Feeder cells treated with low concentration of methanol were unable to adhere on culture dish firmly and most of feeder cells were floated when medium was added back to the dish (Fig. S3). Based on this experiment, 100% methanol was used to fix feeder cells. Meanwhile, the control experiment of incubation of H_2_O with feeder cells was performed. The H_2_O treated cells were unable to be used as feeder cells, showing the similar results as seen in 10% methanol treatment (Fig. S4). The layout of methanol fixation and its applications is presented in Fig. 2A.

Next, we optimized the conditions of methanol-fixed method. The methanol stored at room temperature (RT), 4 °C, and −20 °C, respectively, was used to fix feeder cells, which were then used for culturing J1 cells. There was no influence on J1 mES morphology and AP staining when feeder cells were fixed by methanol in different temperatures (Fig. 2B). Therefore, we selected 4 °C methanol to fix cells. Then, we cultured J1 cells on MT-MEF cells that were fixed for 5 to 20 min at room temperature and found that 5 min treatment was adequate for the fixation of MEFs (Fig. 2C). To optimize the concentration of feeder cells, MEFs from 0.5 × 10^4^/cm^2^ to 3 × 10^4^/cm^2^ were fixed by methanol and used to culture J1 cells. Results of cell morphology and AP staining confirmed that the concentration of feeder cells should be over 1.5 × 10^4^/cm^2^ (Fig. 2D). Too less of feeder cells could influence the pluripotency of stem cells.

To test the storage term of MT-MEF cells, the fixed MEFs were stored at 4 °C (Fig. S5A,B), 37 °C (Fig. S5C,D), and room temperature (Fig. 2E,F) for up to 21 days. The cell morphology and expression level of pluripotent genes indicated that plates coated with MT-MEF cells could be stored for a long term no matter the variation of storage temperatures as long as the fixed cells were dehydrated. Based on above investigations, the optimized condition for methanol fixation is: methanol at 4 °C, fixation of cells for 5 min at RT, and the density of 3 × 10^4^/cm^2^ cells on a plate. Finally, the fixed MEF plate can be stored at RT for at least three weeks.

### Maintenance of self-renewal and pluripotency of mouse PSCs on methanol fixed fibroblasts

**Figure 3:**
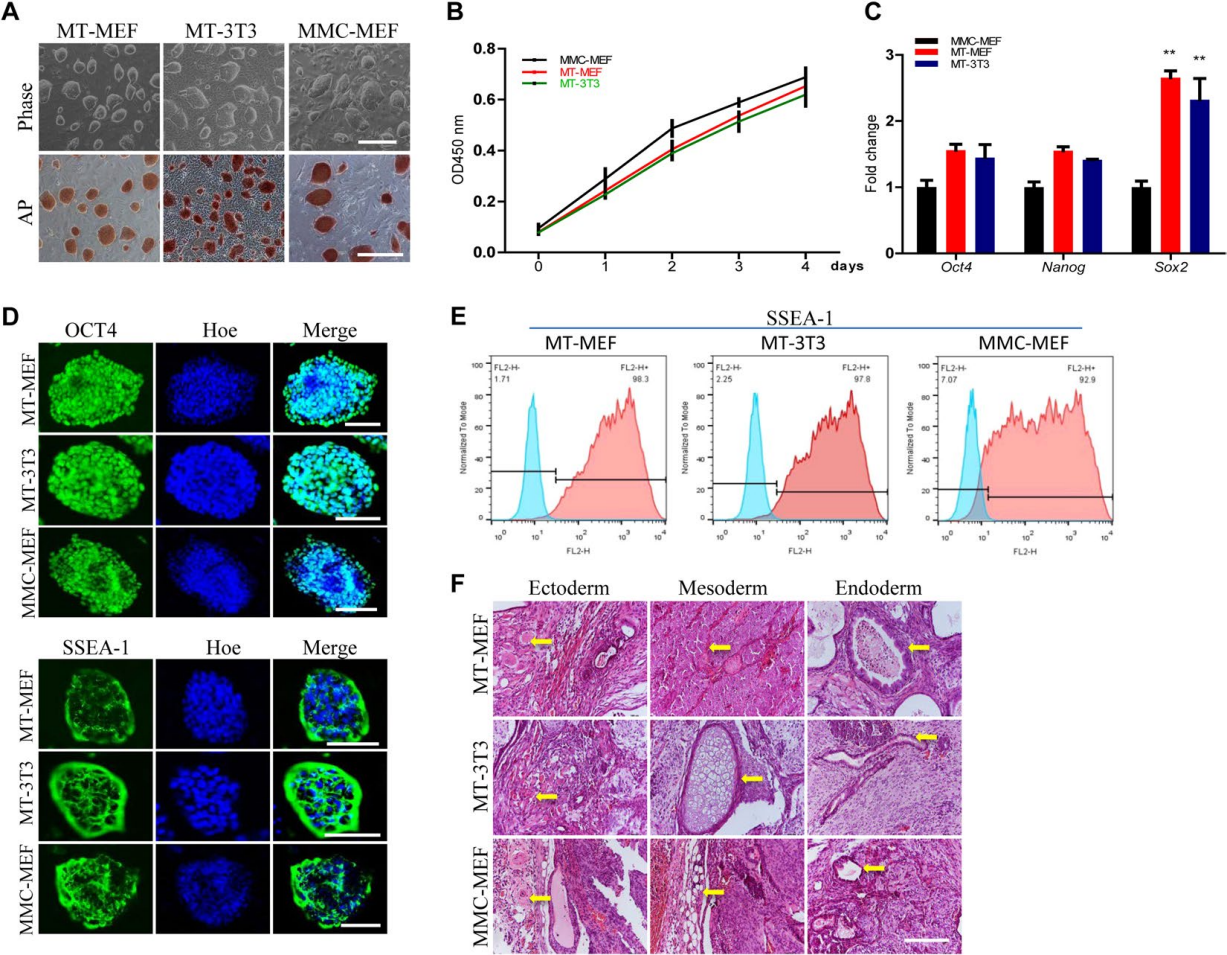
Maintenance of self-renewal and pluripotency of J1 mES on methanol fixed fibroblasts. J1 mES cells were cultured on methanol fixed MEF (MT-MEF) and NIH3T3 (MT-3T3) cells, and Mitomycin C treated MEF (MMC-MEF). (**A**) Morphology and AP staining of J1 mES cells. (**B**) Growth curve of J1 mES. (**C**) qRT-PCR analysis of pluripotent genes in J1 mES. (**D**,**E**) Immunofluorescence (**D**) and flow cytometry analysis (**E**) of pluripotent markers OCT4 and SSEA-1 in J1 mES. Nuclei were stained by Hoechst 33342 (Hoe). (**F**) Teratoma formation of J1 mES. Arrows indicate tissues from the three germ layers. Scale bar, 400 μm for A, 200 μm for F, and 100 μm for D.

Mouse ES J1 and iPS cells were seeded on methanol fixed MEF (MT-MEF) cells, NIH3T3 (MT-3T3) cells, and mitomycin C treated MEF (MMC-MEF) cells, respectively. The morphology and AP activity of J1 and miPS cells cultured on MT-MEF and MT-3T3 were similar to that cultured on MMC-MEF, presenting the compact and domed cell types (Figs 3A, S6A). There was no difference of growth rate of J1 cells on MT-MEF and MT-3T3 versus on MMC-MEF (Fig. 3B). We did qRT-PCR analysis of pluripotent genes using J1 cells that were continuing cultured on MT-MEF for 25 passages and on MT-3T3 for 20 passages, and miPS cells that were continuing cultured on MT-MEF for 20 passages and on MT-3T3 for 18 passages. Results indicated that expression of *Oct4* and *Nanog* from cells on MT-MEF and MT-3T3 was increased versus on MMC-MEF, and *Sox2* expression was significantly upregulated (Figs 3C, S6B). Protein expressions of OCT4 and SSEA-1 in J1 cells, which were continuing cultured on MT-MEF for 30 passages and on MT-3T3 for 25 passages, and miPS cells that were continuing cultured on MT-MEF for 25 passages and on MT-3T3 for 23 passages, were confirmed by immunofluorescence assays (Figs 3D, S6C) and the flow cytometry analysis (Figs 3E, S6D). To further identify the pluripotency of stem cells cultured on MT-MEF and MT-3T3, J1 cells, which were continuing cultured on MT-MEF for 24 passages and on MT-3T3 for 19 passages, and miPS cells, which were continuing cultured on MT-MEF for 15 passages and on MT-3T3 for 13 passages, were carried out the teratoma formation assay. As expected, J1 and miPS cells on MT-MEF and MT-3T3 were able to differentiate into all three germ layers *in vivo* (Figs 3F, S6E). These results indicated that both mES and miPS cells could maintain their self-renewal and pluripotency on methanol fixed fibroblasts for long term passages.

### Growth of human and porcine iPS cells on methanol fixed fibroblasts

**Figure 4:**
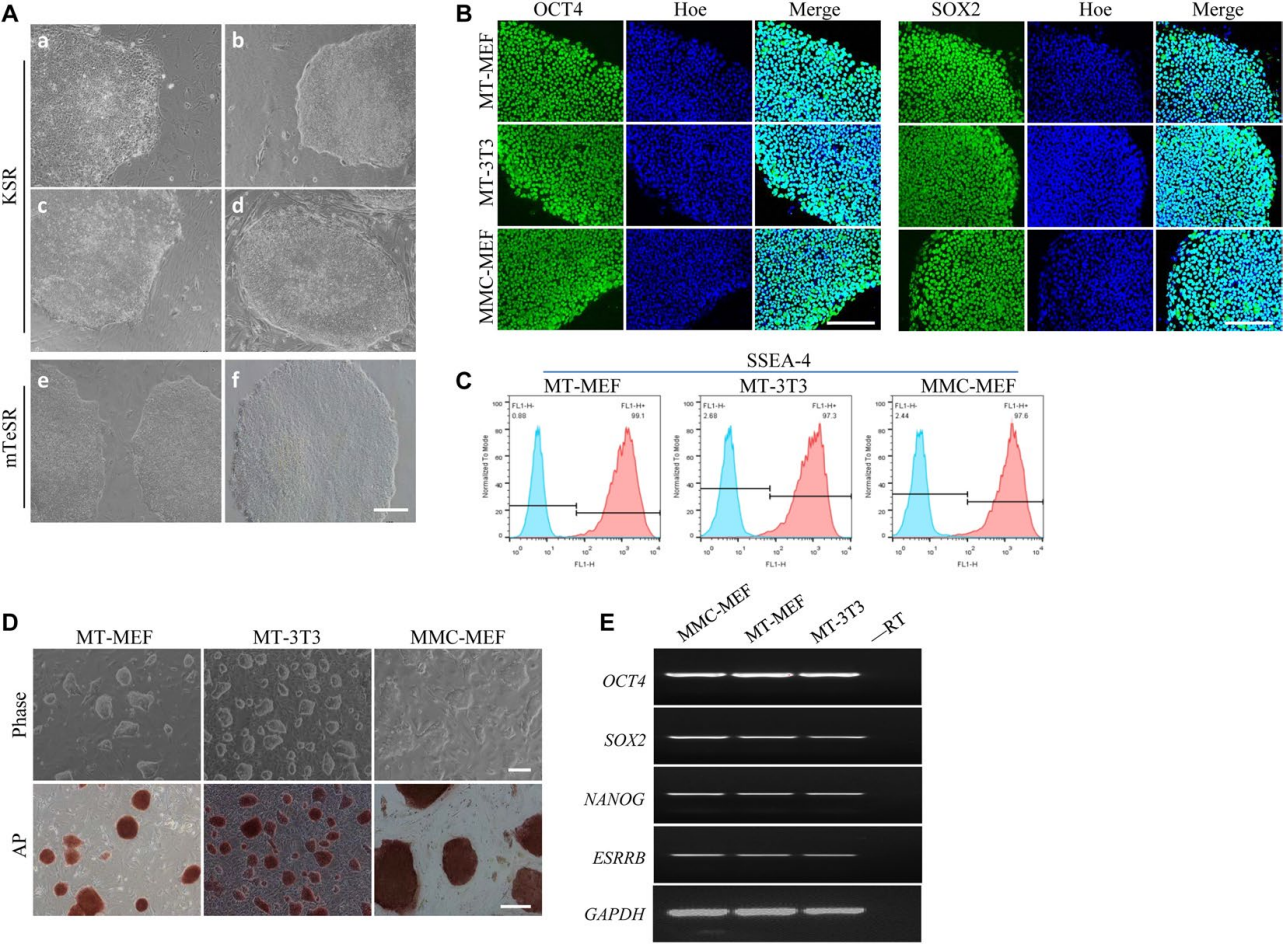
Human and porcine iPS cells were maintained on feeders derived from methanol fixed fibroblasts. (**A**) Human iPS cells were cultured on MT-MEF (a), MT-3T3 (b), MT-hMSC (c), and MMC-MEF (d) in KSR medium, and on MT-MEF (e) and Matrigel (f) in mTeSR medium. (**B**) Immunofluorescence analysis of OCT4 and SOX2 expressions in human iPS cells. (**C**) Flow cytometry analysis of SSEA-4 in human iPS cells. (**D**) Porcine iPS cells were cultured on MT-MEF, MT-3T3, and MMC-MEF. (**E**) RT-PCR analysis of *OCT4*, *SOX2*, *NANOG*, and *ESRRB* expressions in porcine iPS cells. Scale bar, 200 μm.

Commonly, human induced pluripotent stem (hiPS) cells are maintained either on MMC-MEF feeder cells with KSR medium (Fig. 4Ad) or on Matrigel with mTeSR medium (Fig. 4Af)^38,39^. To test whether methanol fixed fibroblasts can be used for culturing human pluripotent stem cells, we cultured hiPS on MT-MEF (Fig. 4Aa), MT-3T3 (Fig. 4Ab), and MT-hMSC (Fig. 4Ac) in KSR medium, and on MT-MEF (Fig. 4Ae) in mTeSR medium for 8 passages. The hiPS morphology did not present any visible changes when cells were cultured on methanol fixed fibroblasts versus on MMC treated MEFs. The pluripotent gene expressions of OCT4 and SOX2 detected by immunofluorescence assay (Fig. 4B) and hiPS specific surface marker SSEA-4 detected by flow cytometry (Fig. 4C) further confirmed that methanol fixed fibroblasts could be used to maintain the pluripotency of hiPS cells.

We also used methanol fixed fibroblasts to maintain porcine induced pluripotent stem (piPS) cells and confirmed that piPS cells cultured on MT-MEF and MT-3T3 retained the typical morphology and positive alkaline phosphatase activity (Fig. 4D). The expression level of pluripotent genes *OCT4*, *SOX2*, *NANOG*, and *ESRRB* of piPS was no change comparing to that on MMC-MEF (Fig. 4E). These observations indicate that methanol fixed fibroblasts can be used not only to maintain mouse PSCs, but also to maintain pluripotent stem cells from other animal species.

### Application of methanol fixed fibroblasts for drug screening and repeated usage

**Figure 5:**
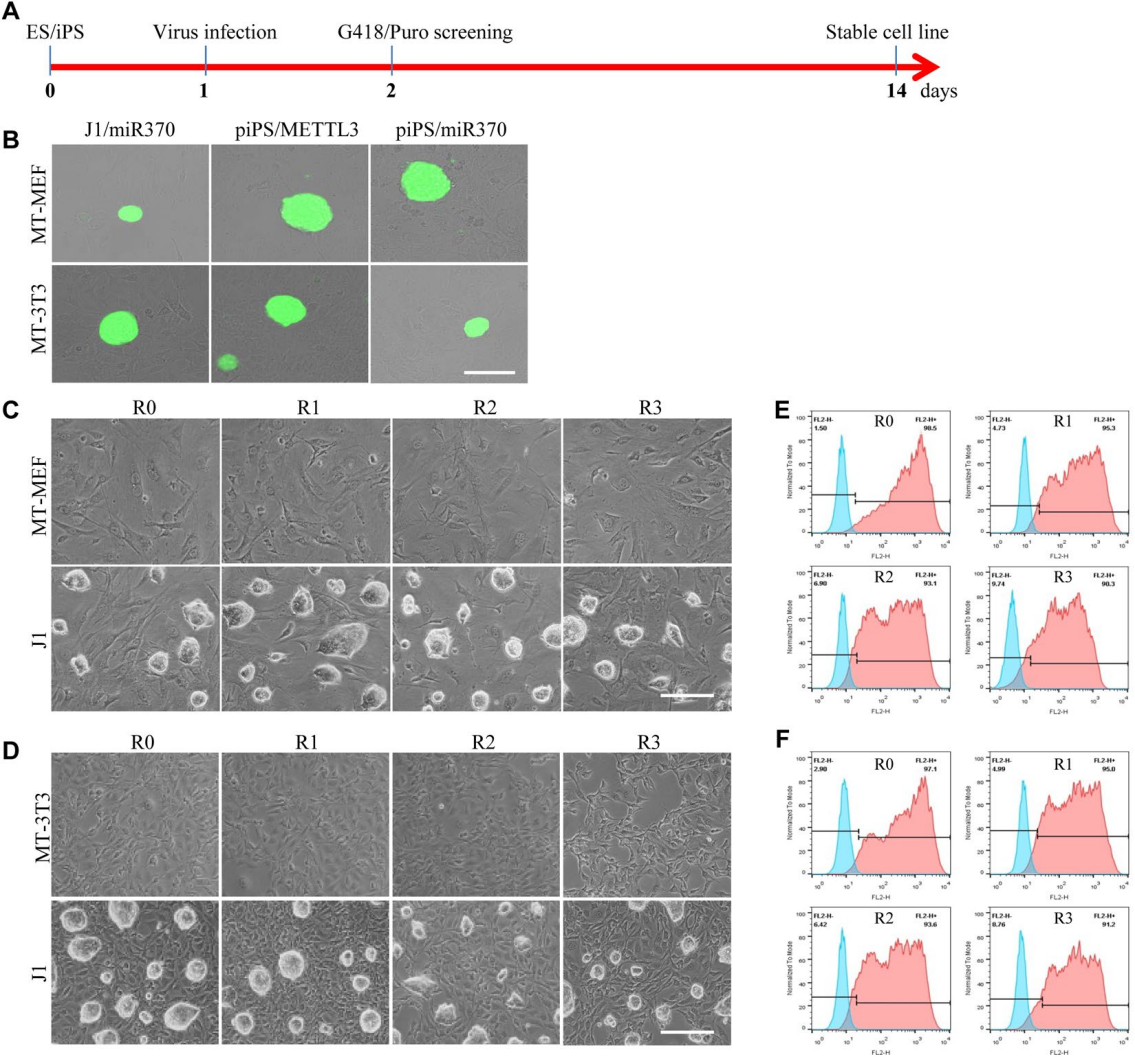
Application of methanol fixed fibroblasts for drug screening and repeated usage. (**A**) Diagram of drug screening. (**B**) The stably transfected cell lines derived from J1 cells transfected by miR70/EGFP and piPS cells transfected by METTL3-EGFP and miR370/EGFP, respectively, which were cultured on MT-MEF and MT-3T3. (**C**,**D**) Repeated usage of MT-MEF (**C**) and MT-3T3 (**D**) for four times (R0-R3). (**E**,**F**) Flow cytometry analysis of SSEA-1 expression in J1 mES cultured on MT-MEF (**E**) and MT-3T3 (**F**), which were used repeatedly for four times (R0-R3). Scale bar, 100 μm for B, 200 μm for C and D.

The mitomycin C treated feeder cells are unable to be used to perform the drug-screen related experiments since the living MEF feeders have the limited tolerance on the high concentration of antibiotic treatment. However, methanol fixed fibroblasts could be used for screening the stable antibiotic-resistant stem cell lines within 14 days (Fig. 5A). We have established several puromycin-resistant stem cell lines that were based on methanol fixed MEF and NIH3T3 cells and through the stable transfection. The puromycin-resistant cell lines include J1 mES cells with miR370 transfection (J1/miR370), and porcine iPS cells with METTL3 transfection (piPS/METTL3) and with miR370 (piPS/miRNA370) (Fig. 5B). Accordingly, the stable antibiotic-resistant pluripotent stem cells can be screened and continuing cultured on methanol fixed fibroblasts.

Since methanol fixed fibroblasts can resist 0.25% Trypsin-EDTA digestion, we asked whether methanol fixed fibroblasts could be repeatedly used to culture pluripotent stem cells. The J1 cells were cultured on the plate coated with MT-MEF (Fig. 5C) and MT-3T3 (Fig. 5D) for 3 days, and then J1 cells were digested by 0.25% Trypsin-EDTA and removed from the plate (R0). The R0-plate washed briefly by PBS was reused for culturing J1 cells again. The plate coated with methanol fixed fibroblasts could be repeatedly used up to 4 times (R1-R3). The flow cytometry analysis showed that SSEA-1 positive J1 cells in R0-plate were 98.5% on MT-MEF (Fig. 5E) and 97.1% on MT-3T3 (Fig. 5F), but SSEA-1 positive J1 cells in R3-plate were dropped to 90.3% on MT-MEF and 91.2% on MT-3T3, indicating that even reused for four times there still were 90 percent J1 cells grown on methanol fixed fibroblasts coated plate. In general, the methanol fixed fibroblasts can be reuse for several times to save time and lower preparation cost.

### Treatment of proteolytic enzymes on methanol fixed fibroblasts

**Figure 6:**
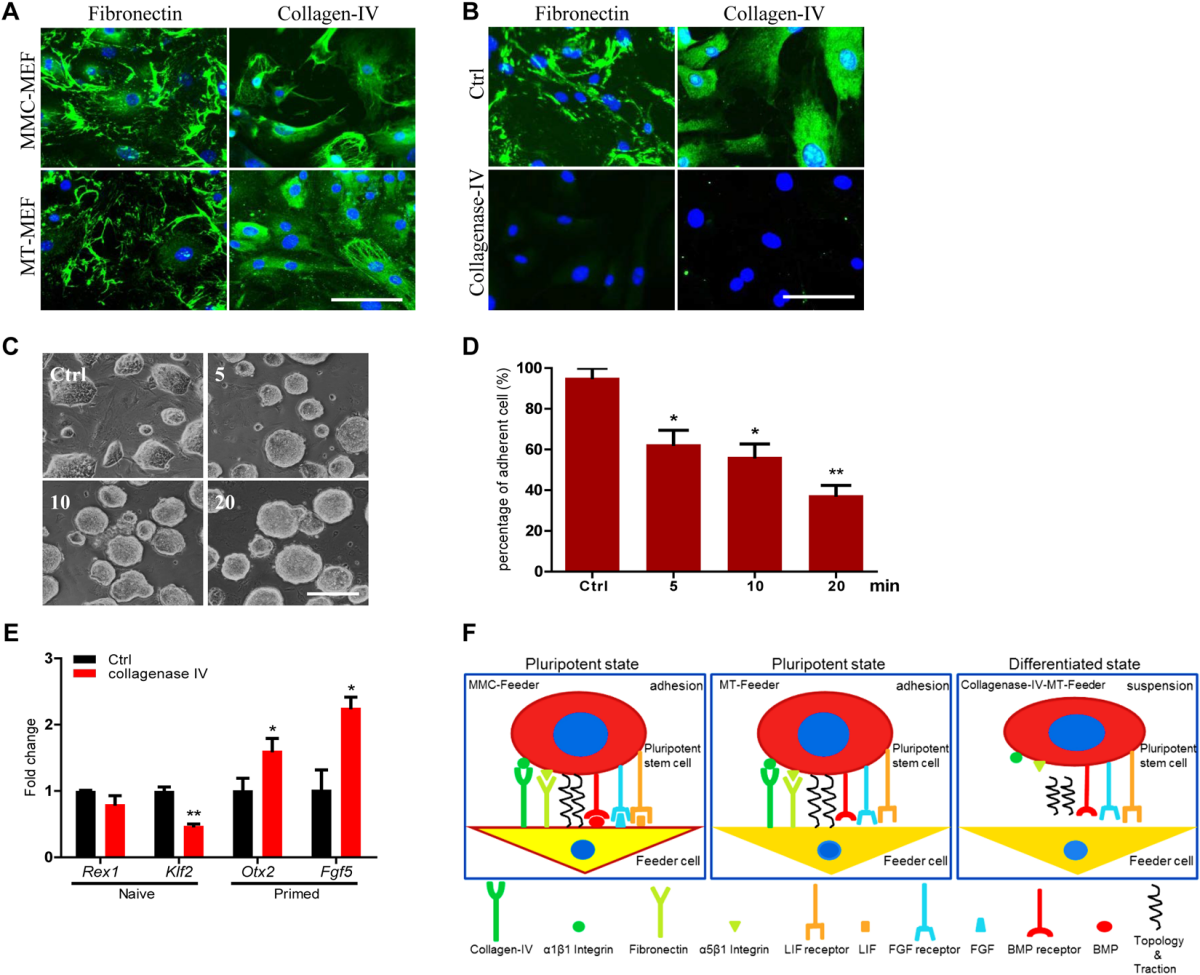
The treatment of proteolytic enzymes on MT-MEF. (**A**) Immunofluorescence analysis of fibronectin and collagen-IV expressions in MT-MEF and MMC-MEF. (**B**) Immunofluorescence analysis of fibronectin and collagen-IV expressions in MT-MEF (Ctrl) and MT-MEF treated by collagenase-IV. (**C**) J1 cells cultured on MT-MEF were treated by collagenase-IV for 5, 10, and 20 min. (**D**) Percentage of J1 cell adherent on MT-MEF treated by collagenase-IV for different times. (**E**) qRT-PCR analysis of expression of naïve and primed markers in J1 cells cultured on MT-MEF (Ctrl) and MT-MEF treated by collagenase-IV. (**F**) Diagram of interaction between different feeder cell niches and pluripotent stem cells. Ctrl, MT-MEF without treatment. Scale bar, 100 μm for A and B, 200 μm for C. Data indicate mean ± SD, *P < 0.05, **P < 0.01, n = 3.

Extracellular matrix (ECM) proteins play major roles on attachment, migration, and signal transduction, and are the key factors to maintain self-renewal and pluripotency of stem cells^34–36^. Though methanol fixed fibroblasts are already dead, pluripotent stem cells can attach tightly and maintain pluripotency state well. We hypothesized that ECM proteins were still existed in the surface of methanol fixed fibroblasts. Immunofluorescence assay confirmed that ECM proteins fibronectin and collagen-IV were in MEF (Fig. 6A) and NIH3T3 (Fig. S7A) cells treated with methanol.

Collagenase-IV is a metalloprotease that degrades ECM proteins such as collagen-IV and fibronectin^37^. We identified that collagen-IV and fibronectin were degraded from MT-MEF (Fig. 6B) and MT-3T3 (Fig. S7B) cells, which were treated by collagenase-IV for 20 min. As long as 5-min collagenase-IV treatment on MT feeder cells, J1 cells were unable to grow well and many colonies were suspended (Figs 6C, S7C). The number of adherent colonies was reduced significantly on MT-MEF (Fig. 6D) and MT-3T3 (Fig. S7D) feeders that were treated by collagenase-IV for 20 min. The gene expression of J1 cells cultured on MT-MEF treated by collagenase-IV for 20 min showed that naïve state genes *Rex1* and *Klf2* were reduced and primed state genes *Otx2* and *Fgf5* were increased comparing with J1 cultured on MT-MEF (Fig. 6E). These results demonstrated that ECM proteins collagen-IV and fibronectin were crucial for adhesion and naïve state maintenance of PSCs (Fig. 6F).

## Discussion

Normally, ES and iPS cells were cultured on mitomycin C or gamma radiation treated MEF feeder cells^4–6^. Preparation of MEF feeder cells needs the primary embryonic fibroblasts that are isolated from E12.5 mouse embryo and expanded *in vitro* for one to three passages^12,13^. Therefore, making MEF feeder cells is time consuming and involved in multiple processes that may lead to the cross contaminations. Using chemical fixer GA and FA to fix feeder cells provides an alternative approach to produce MEF feeders and maintain pluripotency of PSCs^31–33^. The low concentration of FA/GA was able to sustain the membrane fluidity that was correlated to the biological activity to maintain PSCs^40^. In this study, we found that methanol could also be as the chemical fixer to straightforwardly make MT-MEF feeder cells, which is very maneuverable and reproducible, and also the plate with methanol fixed feeder cells is able to be stored at room temperature for a long term and to be reused for multiple times. We also demonstrated that ECM proteins in the surface of methanol fixed cells were important for the adhesion of stem cells.

There are several great advantages of methanol fixed feeder cells versus the traditional mitomycin C treated MEF feeder cells. First, since methanol fixed fibroblasts can be stored at room temperature for a long term (up to 21 days), plates with methanol fixed feeder cells can be made in large scale once and for all, saving time and reducing costs. Second, unlike mitomycin C treated MEFs, methanol fixed cells can theoretically provide a germ-free feeder and significantly reduce the cross contaminations because all proteins inside and outside of cells have been fixed by methanol. Third, unless MEFs were previously transfected by drug-resistant gene, mitomycin C treated MEFs cannot be used to perform drug-resistant screening assays, instead, methanol fixed fibroblasts could be directly used for drug screening experiments. Furthermore, we found that besides MEFs the commercial cell lines, including fibroblast NIH-3T3, myoblast C2C12, and porcine kidney epithelium PK-15 cells, could be fixed by methanol and used as feeder cells. The PSCs cultured on MT-3T3 feeders were maintained the pluripotency similar to that on MT-MEF and MMC-MEF. This discovery extremely extends the applications of methanol fixation method and has the potential for the commercial products.

In addition to the traditional MEF feeder cells, more and more feeder-free culturing systems for PSCs were developed, which include Matrigel^19^, laminin^21^, recombinant fibronectin^20^, vitronectin^22^, and 3D-hydrogel^25,26^ were specially used to culture human PSCs, providing a xeno-free condition for the expansion and clinical application of hPSCs. However, the culture dish coated by artificial matrixes is pricey and needs particular media and growth factors. Therefore, feeder-free culturing systems are limited for the large-scale preparation of PSCs. Alternatively, the methanol fixation method is simple and economical, which can be easily used for large scale preparation of PSCs.

The reasons why pluripotent stem cells should be cultured on feeder cells to maintain their undifferentiated state were not clear so far. Previously, we and others thought that mitomycin C and radiation treated MEF feeder cells were able to produce and secret growth factors and cytokines to provide an environment to maintain pluripotency of PSC^9–11^. However, in this study, we found that MEF feeder cells were died after be fixed by methanol and couldn’t produce growth factors and cytokines supporting PSC growth. Consequently, growth factors and cytokines that secret from MEF feeder cells are not necessary for maintaining PSCs. On the other hand, extracellular matrix proteins in the surface of feeder cells were reported to play important role in cell attachment and maintaining pluripotency of PSCs^34–36^. We detected extracellular matrix proteins collagen-IV and fibronectin in methanol fixed fibroblasts and confirmed that they exist in fibroblasts even after be treated by methanol. Besides, the significance of topology^41–43^ and traction^44–46^ between pluripotent stem cells and their adhesion surface was reported in recent years. Our experiments demonstrated that ECM proteins were crucial in adhesion and pluripotency to maintain PSC. In this study, we only detected two ECM proteins collagen-IV and fibronectin, whether other ECM proteins also played role in adhesion for PSCs were not known yet. We hypothesis that the role of feeder cells for PSC is the joint action including ECM proteins, cell adhesion molecules, topology, traction and others that may not be investigated yet.

## Conclusions

In summary, our novel method can substitute traditional method for making feeder cells completely. In addition to MEF cells, commercial cell lines including immortalized fibroblasts, myoblasts, and epithelium cells can be fixed by methanol and used to culture pluripotent stem cells. Not only mouse PSCs, but also PSCs from other species can be cultured on methanol fixed fibroblasts. Methanol fixed fibroblasts can be reused and used for screening antibiotic selection to establish stable pluripotent stem cell lines. We also demonstrate that ECM proteins collagen-IV and fibronectin play a crucial role for adhesion and maintaining pluripotency of PSCs.

## Methods

### Animals

The usage of live ICR mice for making mouse embryonic fibroblasts (MEFs) in this study was approved by Animal Care and Use Committee, which was subjected to Experiment Manage Committee of Northwest A&F University. The experimental protocols that are involved in animal samples were based on the guideline approved by the Animal Research Committee of Northwest A&F University. The nude mice used for teratoma experiments and tissue sections were conducted and paid in the Experimental Animal Center of Fourth Military Medical University, which has the licensed animal research facility and provides the commercial services. All methods were carried out in accordance with relevant guidelines and regulations.

### Preparation of feeder cells

To prepare feeders, MEF cells were cultured on culture dish with MEF medium (DMEM high glucose plus 15% FBS) to 80–90% confluence and then 10 μg/mL mitomycin C was added. After 2.5 h incubation at 37 °C, mitomycin C was removed and cells were washed with phosphate buffer saline (PBS) for 3 times. Cells were digested with 0.25% Trypsin-EDTA and then be used immediately or stored in liquid nitrogen. To prepare glutaraldehyde (GA) fixed MEF feeder, cells were cultured in culture dish with MEF medium to 80–90% confluence and then fixed with 2.5% glutaraldehyde as reported previously^31–33^. To prepare methanol fixed feeders, MEFs were cultured with MEF medium and NIH3T3 cells were cultured with NIH3T3 medium (DMEM high glucose plus 10% FBS). When cells were in 80–90% confluence, media were removed and cells were washed with PBS once. The 100% methanol precooled at 4 °C was added into the dish for 5 min at room temperature. After removing methanol, the dish was opened and placed on the surface of flow hood clean bench for 5 min to make sure that methanol was fully volatilized, and the fixed cells were dehydrated. Cells treated by methanol are ready to be used as feeders immediately or be stored at room temperature for future use. As the control experiment, cells were treated by H_2_O for 5 min at room temperature, and then following the similar steps of methanol treatment.

### Expansion of mES and miPS on methanol-fixed fibroblasts

J1 mES cells were purchased from ATCC and miPS cells were induced by our lab colleagues. J1 mES and miPS cells were digested into single cells with 0.25% Trypsin-EDTA and then seeded on methanol-fixed MEF and NIH3T3 feeders in normal mES medium including DMEM high glucose supplemented with 15% FBS, 0.1 mM non-essential amino acids (NEAA, Gibco, USA), 1 mM L-glutamine (Gibco, USA), 0.1 mM β-mercaptoethanol (β-met, Sigma, USA) and 10^3^ units/mL mLIF (ESG1107, Millipore). Media were changed every two days.

### Human and porcine iPS cultured on methanol-fixed MEF and NIH3T3 cells

Human iPS cells were purchased from SiDan Sai Biotechnology Co., Ltd. and porcine iPS cells were induced by our lab colleagues with Tet-On system. The hiPSCs were seeded on methanol-fixed MEF and NIH3T3 feeder cells, and cultured with DMEM/F12 supplemented with 20% KSR (Gibco, USA), 0.1 mM NEAA, 1 mM L-glutamine, 0.1 mM β-met and 10 ng/mL bFGF (100-18B, PeproTech) or with mTeSR^TM^1 medium (#85850, STEMCELL Technologies). The piPSCs were seeded on methanol-fixed MEF and NIH3T3 feeder cells with DMEM supplemented with 15% FBS, 0.1 mM NEAA, 1 mM L-glutamine, 0.1 mM β-met, 10 ng/mL hLIF (LIF1050, Millipore) and 10 ng/mL bFGF (100-18B, PeproTech). Media were changed every two days.

### RT-PCR

Total RNAs were extracted by Trizol Reagent (Thermo Fisher Scientific, USA) according to the manufacturer’s procedure. RNAs were examined by measuring OD260/280 ratio, and samples with a ratio of 2.0 were used for reverse transcription. One microgram total RNAs were reverse-transcribed with oligo-dT primer (Thermo Fisher Scientific, USA) using RevertAid^TM^ reverse transcriptase (Thermo Fisher Scientific, USA). RT-PCRs were performed using 2 × Es Taq MasterMix (CW Biotech, China) for 30 cycles at 94 °C 30 s, 58 °C 30 s, and 72 °C 30 s. Non-RT negative controls (RT−) were also performed to monitor non-specific reactions, and *Gapdh* was used as internal controls. Quantitative RT-PCRs (qRT-PCR) were performed using a 10-fold dilution of cDNA with SYBR Green PCR Master Mix (TRANSGEN BIOTECH, China), and detected with StepOnePlus Real-Time PCR System (Applied Biosystems, USA). Measurements were performed on three biological replicates and each reaction was performed in triplicate. The expression level of target gene was normalized to the expression level of *Gapdh*. Melting curve analysis was conducted to confirm the specificity. Primers used in this study are listed in Supplementary Table S1.

### Alkaline phosphatase staining

The alkaline phosphatase (AP) activity of pluripotent stem cells was determined by AST Fast Red TR and α-Naphthol AS-MX Phosphate (Sigma Aldrich, USA) according to the manufacturer’s instructions. Briefly, cells were washed twice using ice-cold PBS, fixed with 4% paraformaldehyde in PBS (pH 7.4) for 15 min at room temperature, followed by washing three times using ice-cold PBS. Cells were then incubated at room temperature in the solution containing 1.0 mg/mL Fast Red TR, 0.4 mg/mL α-Naphthol AS-MX in 0.1 M Tris buffer. After 5–10 min incubation, AP positive colonies were displayed in red.

### Growth curve

CCK-8 cell counting kit (A311-01, Vazyme, China) was used to calculate growth rate of J1 mES and miPS cells cultured on methanol fixed MEF and NIH3T3 cells along with MMC-MEF feeder cells according to the manufacturer’s procedure. 2 × 10^3^ J1 or miPS cells were seeded on methanol fixed cells in 48 well plates for 12 h. The MMC-MEF feeders were used as control. Then, 20 uL CCK-8 regent was added per well and incubated at 37 °C for 1 h. The absorbance OD values at 450 nm were then measured as Day 0.

### Immunofluorescence

For immunofluorescence assays, cells were fixed with 4% paraformaldehyde for 15–30 min at room temperature. The fixed cells were washed twice with PBS, incubated with PBS containing 0.1% Triton X-100 for 10 min, and washed three times with PBS. After blocking in BSA-blotting buffer (1% BSA and 0.1% Tween 20 in PBS) for 30 min, cells were incubated in BSA-blotting buffer with primary antibodies, including anti-Oct4 (1:200, sc-5279, Santa Cruz), anti-Sox2 (1:200, sc-365823, Santa Cruz), anti-Nanog (1:200, ab80892, Abcam), anti-SSEA-1 (1:200, 4744 S, Cell Signaling Technology), anti-Fibronectin (1:100, 15613-1-AP, Proteintech) and anti-Collagen-IV (1:50, 19797-1-AP, Proteintech), in a humidified chamber at 4 °C overnight. After washing three times, cells were stained for 1 h with either anti-mouse or anti-rabbit secondary antibody (1:200, Proteintech, China). Nuclei were stained with Hoechst33342 (10 μg/mL) for 2–5 min. Microscopy was performed on a Leica fluorescence microscope.

### Flow cytometry analysis

For flow cytometry analysis, pluripotent stem cells were washed once with PBS and then detached with Accutase (Gibco, USA). After centrifugation, cells were washed twice with PBS and resuspended in stain buffer (PBS with 2% FBS) for cell counting. 1 × 10^6^ cells were then transferred into separate 1.5 ml Eppendorf tubes and incubated in 100 μL staining buffer supplemented with FITC-anti-SSEA-1 (#560886, BD pharmingem, USA) or PE-anti-SSEA-4 antibody (#330409, Biolegend, USA) for 30 min on ice, protected from light. Cells were washed twice and resuspended in 500 μL staining buffer and analyzed with FACSCalibur (BD Biosciences). FACS Data were analyzed with FlowJo software.

### Scanning Electron Microscope

MEF cells were seeded on round cover slips and cultured to 80% confluence. After be treated with methanol, 2.5% glutaraldehyde and MMC, cells were dehydrated stepwise by 10%, 30%, 50%, 70%, 80%, and 90% ethanol for 10 min. After drying, sticking, metal spraying, samples were observed with Hitachi S4800 Scanning Electron Microscope.

### Teratoma formation

Total of 6 samples, including J1-MT-MEF-P30, J1-MT-3T3-P20, J1-MMC-MEF and miPS-MT-MEF-P29, miPS-MT-3T3-P20, miPS-MMC-MEF, were harvested and injected subcutaneously into dorsal flanks of immunodeficient mice (5 × 10^6^ cells per injection site). Control mice received 1xPBS only. After 4 weeks, the nude mice were sacrificed and the teratomas were collected, fixed with 4% paraformaldehyde, and tissues were stained with Hematoxylin and Eosin as the previous description^47,48^. Images were obtained under a Nikon microscope.

### Antibiotic-resistant assay

To screening the stable pluripotent stem cell lines, lentiviral vectors pCDH-*METTL3*-copGFP-Puro and pCDH-miR370-copGFP-Puro were constructed for packing virus. To make lentiviral particles, 2 × 10^6^ HEK-293T cells were seeded on a 60 mm cultural dish. When cells reach to 80% confluence, 4 μg each of pCDH-*METTL3*-copGFP-Puro and pCDH-miR370-copGFP-Puro vectors were transfected into HEK-293T cells together with 4 μg pCL-Eco and 2 μg pCMV-VSV-G at a ratio of 2:2:1 using Lipofectamine 2000 (Invitrogen). After 48 h post-transduction, media with viral particles were collected and filtered through 0.45 μm filter (Millipore). The collected virus suspension mixed with 8 μg/mL Polybrene were infected into J1 mES and piPS cells. J1 mES cells were cultured in medium with 2 μg/mL puromycin and piPS were cultured in medium with 4 μg/mL puromycin for 1 weeks. The puromycin-resistant colonies of J1 mES and piPS cells were then picked up and expanded in the medium with 2 μg/mL puromycin.

### Statistical Analysis

Values were presented as the mean ± SD. Statistical analyses were performed with SPSS. Two-way ANOVA were used to study differences between grouped data, Student’s T tests were performed with one way analysis. Statistical significance was accepted at *P* < 0.05. All flow cytometry data were analyzed and generated by FlowJo software.

### Data availability

All data generated or analyzed during this study are included in this published article (and its Supplementary Information files).

## Electronic supplementary material

supplementary information
