# Supplementary material for: Methanol fixed fibroblasts serve as feeder cells to maintain stem cells in the pluripotent state in vitro
Source: Sci Rep. 2018 May 17;8:7780. doi: 10.1038/s41598-018-26238-2 (PMC5958091; doi:10.1038/s41598-018-26238-2)
Supplement: Supplementary file 1 — supplementary information [file 41598_2018_26238_MOESM1_ESM.pdf]

Supplementary Information

Methanol fixed fibroblasts serve as feeder cells to maintain stem cells in the pluripotent state in vitro

Running title: Maintenance of PSCs on methanol-fixed feeder cells

Yahui Ren[1], Ziyu Ma[1], Tong Yu[1], Min Ling[2], and Huayan Wang[1]*

[1]Department of Animal Biotechnology, College of Veterinary Medicine, Northwest A&F University, Yangling, Shaanxi 712100, China

[2]Department of Innovation Experimental College, Northwest A&F University, Yangling, Shaanxi 712100, China

* Correspondence and requests for materials should be addressed: Prof. Huayan Wang, Department of Animal Biotechnology, College of Veterinary Medicine, Northwest A&F University, Yangling, Shaanxi 712100, China.

Telephone: 86-029-87080069; Fax：86-029-87080068; E-mail: hhwang101@163.com or hhwang101@nwafu.edu.cn.

Supplementary Table

Table S1 Primers used in this study

Name	Sequence (5'-3')	Size (bp)	Accession No
mc-q-*Nanog*	F:TCTTCCTGGTCCCCACAGTTT<br>R:GCAAGAATAGTTCTCGGGATGAA	100	NM_028016
mc-q-*Oct4*	F:CTCGAACCTGGCTAAGCTTCC<br>R:CATCCCTCCGCAGAACTCGT	113	NM_013633
mc-q-*Sox2*	F:ACTCCATGACCAGCTCGCAGA<br>R:CTGGCCTCGGACTTGACCAC	124	NM_011443
mc-q-*Otx2*	F:TATCTAAAGCAACCGCCTTACG<br>R:AAGTCCATACCCGAAGTGGTC	62	NM_144841
mc-q-*Klf2*	F:TCGAGGCTAGATGCCTTGTGA<br>R:AAACGAAGCAGGCGGCAGA	133	NM_008452.2
mc-q-*Rex1*	F:CCCTCGACAGACTGACCCTAA<br>R:TCGGGGCTAATCTCACTTTCAT	112	NM_009556
mc-q-*Fgf5*	F:GCTGTGTCTCAGGGGATTGT<br>R:CACTCTCGGCCTGTCTTTTC	191	NM_010203
mc-q-*Gapdh*	F:AGGTCGGTGTGAACGGATTTG<br>R:TGTAGACCATGTAGTTGAGGTCA	123	NM_008084.2
pc-RT-*OCT4*	F:TGAGGCTTTGCAGCTCAGTT<br>R:ACTGCTTGATCGTTTGCCCT	310	NM_001113060
pc-RT-*SOX2*	F:CATGAAGGAGCACCCGGATT<br>R:CCGTTCATGTAGGTCTGCGA	354	NM_001123197
pc-RT-*NANOG*	F:TTGCCCCGAAGCATCCATT<br>R:CCAGCTCTGATTACCCCACA	565	NM_001129971
pc-RT-*ESRRB*	F:TGAGATCACCAAACGGAGGC<br>R:GAGAAGCCTGGGATGTGCTT	360	XM_001928051
pc-RT-*GAPDH*	F:AAGGTCGGAGTGAACGGATT<br>R:AGTCTTCTGGGTGGCAGTGAT	549	NM_001206359.1

mc: mouse, pc: porcine, q: qRT-PCR, RT: RT-PCR.

Supplementary Figure legends

Figure S1. Morphology and AP staining of J1 mES cultured on different types of matrix. Scale bar, 200 μm.

Figure S2. MEFs fixed by methanol and acetone in different proportions. (A) Morphology and AP staining of J1 mES cultured on MEFs fixed by methanol (MT) and acetone (AT) in different proportion. **(B)** qRT-PCR analysis of *Oct4* and *Nanog* expressions in J1 mES. **(C)** The percentage of AP positive colonies of J1 mES. Ctrl, MEFs were treated by mitomycin C. Phase 1, morphology of MEFs; Phase 2, morphology of J1 mESCs. Scale bar, 400 μm.

Figure S3. Feeder cells fixed by different concentration of methanol. MEFs **(A)** and NIH3T3 **(B)** were fixed by methanol in different concentration (10% to 100%). Phase 1, methanol fixed feeder cells; Phase 2, the dehydrated methanol-fixed feeder cells; Phase 3, the methanol-fixed feeder cells in culture medium ready for seeding stem cells. Scale bar, 100 μm.

Figure S4. Feeder cells treated by H_2O and methanol. MEFs **(A)** and NIH3T3 **(B)** cells were treated by H_2O for 5 min. Methanol-fixed cells were as control. Phase 1, H_2O and methanol treated feeder cells; Phase 2, the dehydrated H_2O treated feeder cells; Phase 3, the H_2O and methanol treated feeder cells in culture medium ready for seeding stem cells. Scale bar, 100 μm.

Figure S5. Storage of MT-MEFs. J1 mESCs were cultured on MT-MEFs that were stored for different dates and in the different temperatures. **(A)** MT-MEFs were stored at 4°C for 0 to 21 days. **(B)** qRT-PCR analysis of *Oct4* and *Nanog* expressions in J1 mES on MT-MEF that

was stored at 4°C for different dates. **(C)** J1 mES cultured on MT-MEF stored at 37°C for 0 to 21 days. **(D)** qRT-PCR analysis of *Oct4* and *Nanog* expressions in J1 mES cultured on MT-MEF that was stored at 4°C for different dates. Scale bar, 200 μm.

Figure S6. Methanol treated fibroblasts can maintain self-renewal and pluripotency of mouse iPS cells. **(A)** Mouse iPS cells were cultured on MT-MEF cells, MT-3T3 cells, and mitomycin C treated MEF (MMC-MEF). **(B)** qRT-PCR analysis of pluripotent genes in miPS cells cultured on the different feeders. **(C-D)** Immunofluorescence (C) and flow cytometry (D) analyses of pluripotent markers OCT4 and SSEA-1in miPS cells cultured on the different feeders. **(E)** Teratoma formation of miPS cells cultured on the different feeders. Arrows indicate tissues from the three germ layers. Scale bar, 400 μm for A, 100 μm for B, 200 μm for E.

Figure S7. The treatment of proteolytic enzymes on MT-3T3. **(A)** Immunofluorescence analysis of fibronectin and collagen-IV expressions in MT-3T3 and normal NIH3T3 (control). **(B)** Immunofluorescence analysis of fibronectin and collagen-IV expressions in MT-3T3 (control) and MT-3T3 treated by collagenase-IV. **(C)** Morphology of J1 cells cultured on MT-3T3 treated by collagenase-IV from 5, 10, and 20 min. **(D)** Percentage of J1 cell adherent on collagenase-IV treated MT-3T3 for different times. Scale bar, 100 μm for A and B, 200 μm for C. Data indicate mean ± SD, * $P < 0.05$, ** $P < 0.01$, n =3.

Figure S1

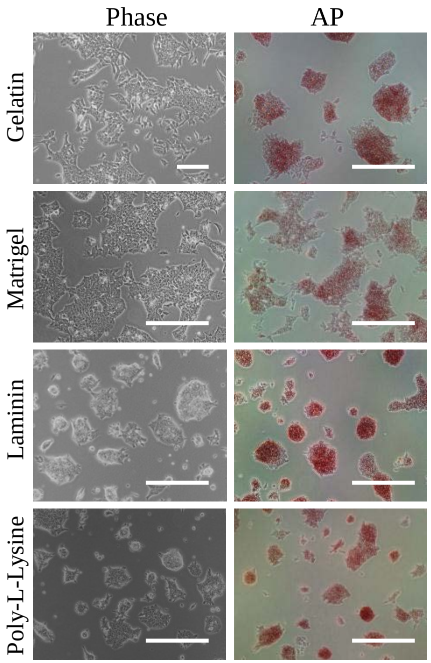

Figure S2

A

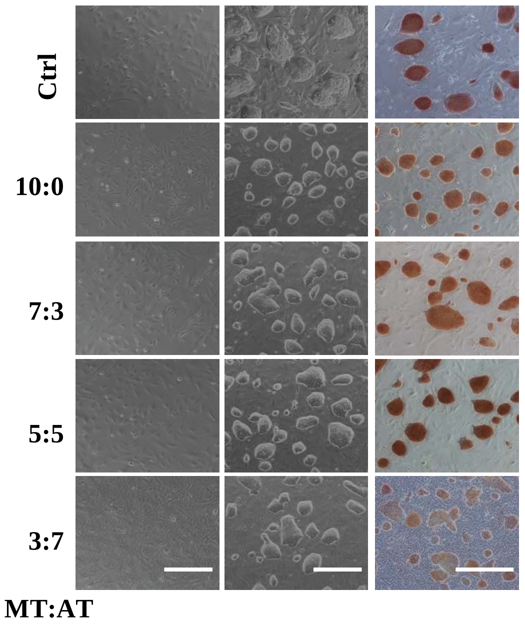

B

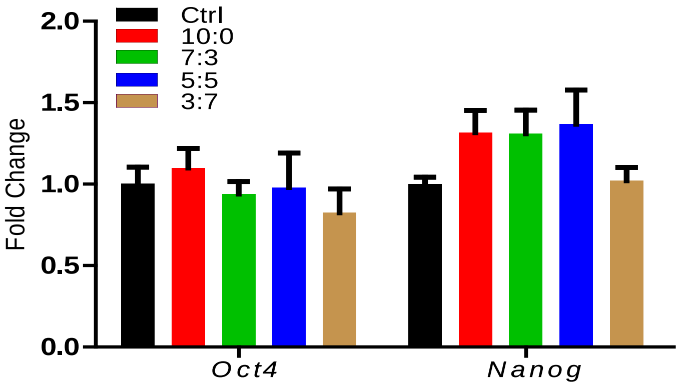

C

AP Positive rate (%)				
MMC	10:0	7:3	5:5	3:7
98.22 ± 2.07	97.78 ± 0.58	97.80 ± 0.75	98.12 ± 0.5	90.41 ± 2.63

Figure S3

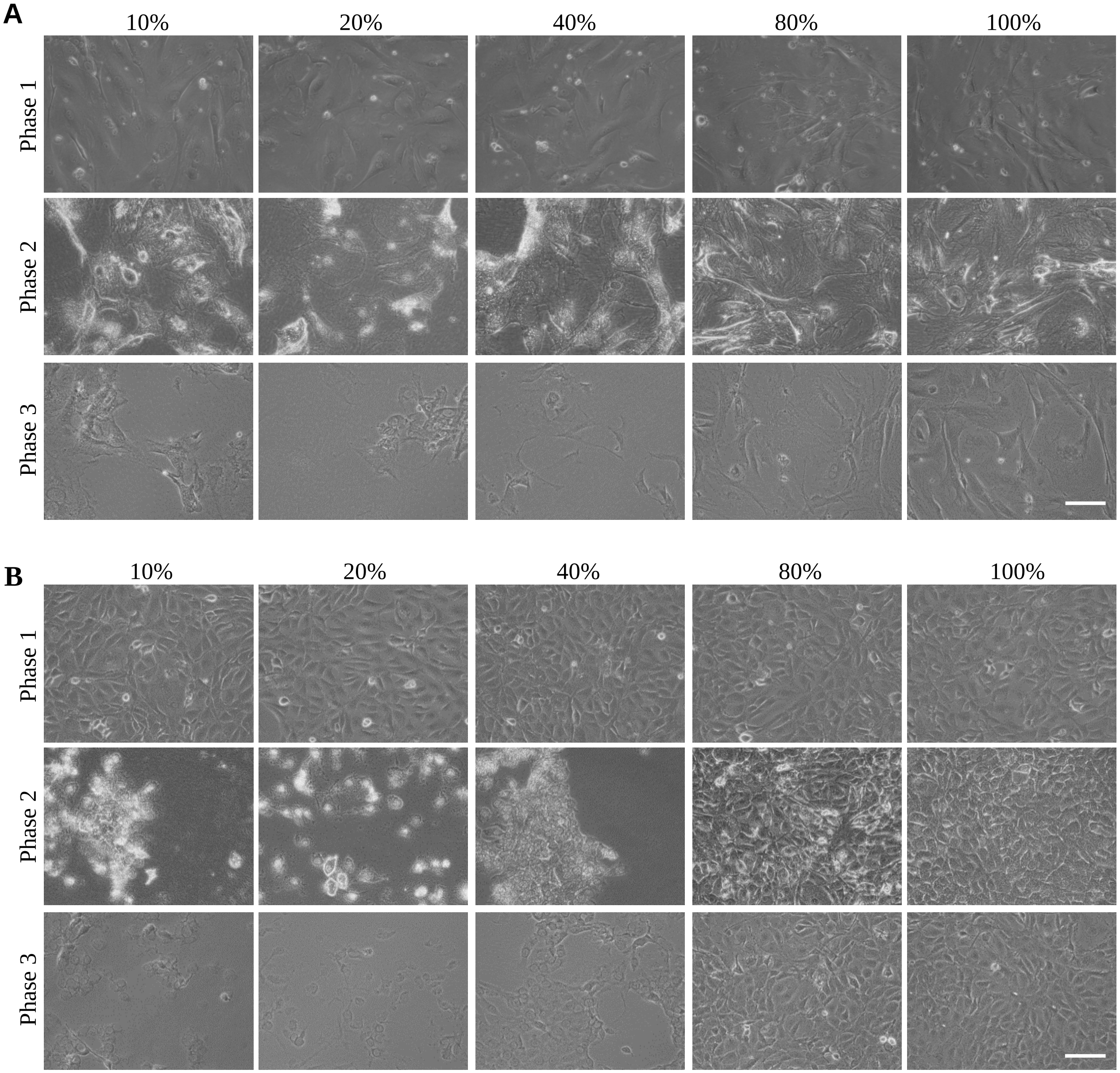
A
10%
20%
40%
80%
100%
Phase 1
Phase 2
Phase 3
B
10%
20%
40%
80%
100%
Phase 1
Phase 2
Phase 3

Figure S4

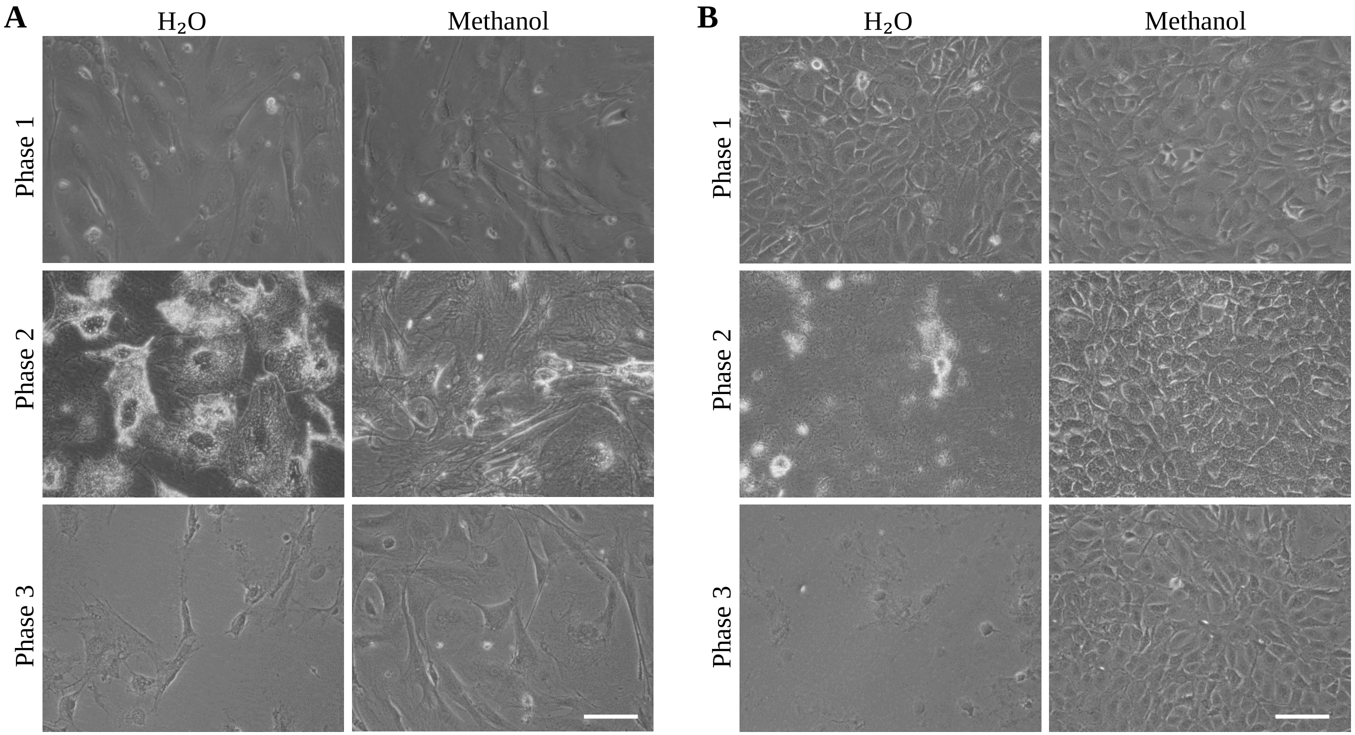
A
B
H2O
Methanol
H2O
Methanol
Phase 1
Phase 2
Phase 3
Phase 1
Phase 2
Phase 3

Figure S5

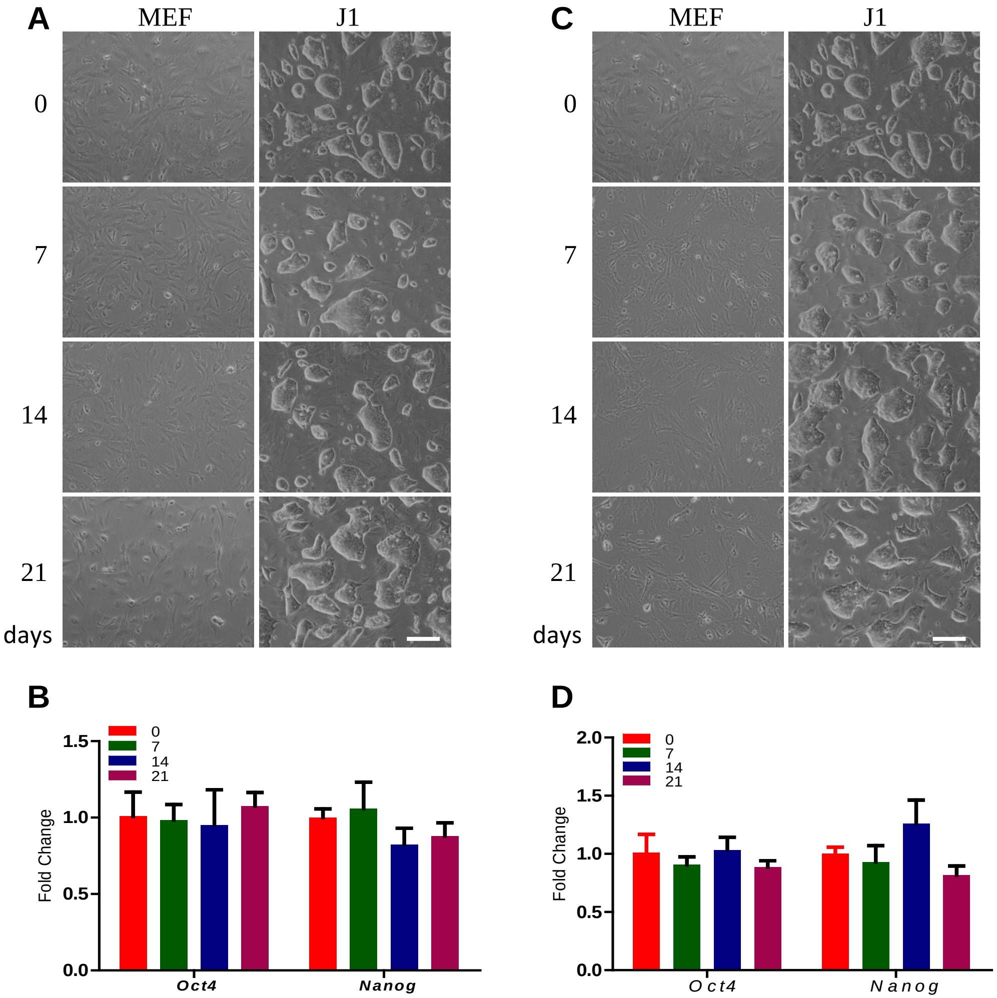
A
MEF
J1
0
7
14
21
days
C
MEF
J1
0
7
14
21
days
B
0
7
14
21
1.5
1.0
0.5
0.0
Fold Change
Oct4
Nanog
D
0
7
14
21
2.0
1.5
1.0
0.5
0.0
Fold Change
Oct4
Nanog

Figure S6

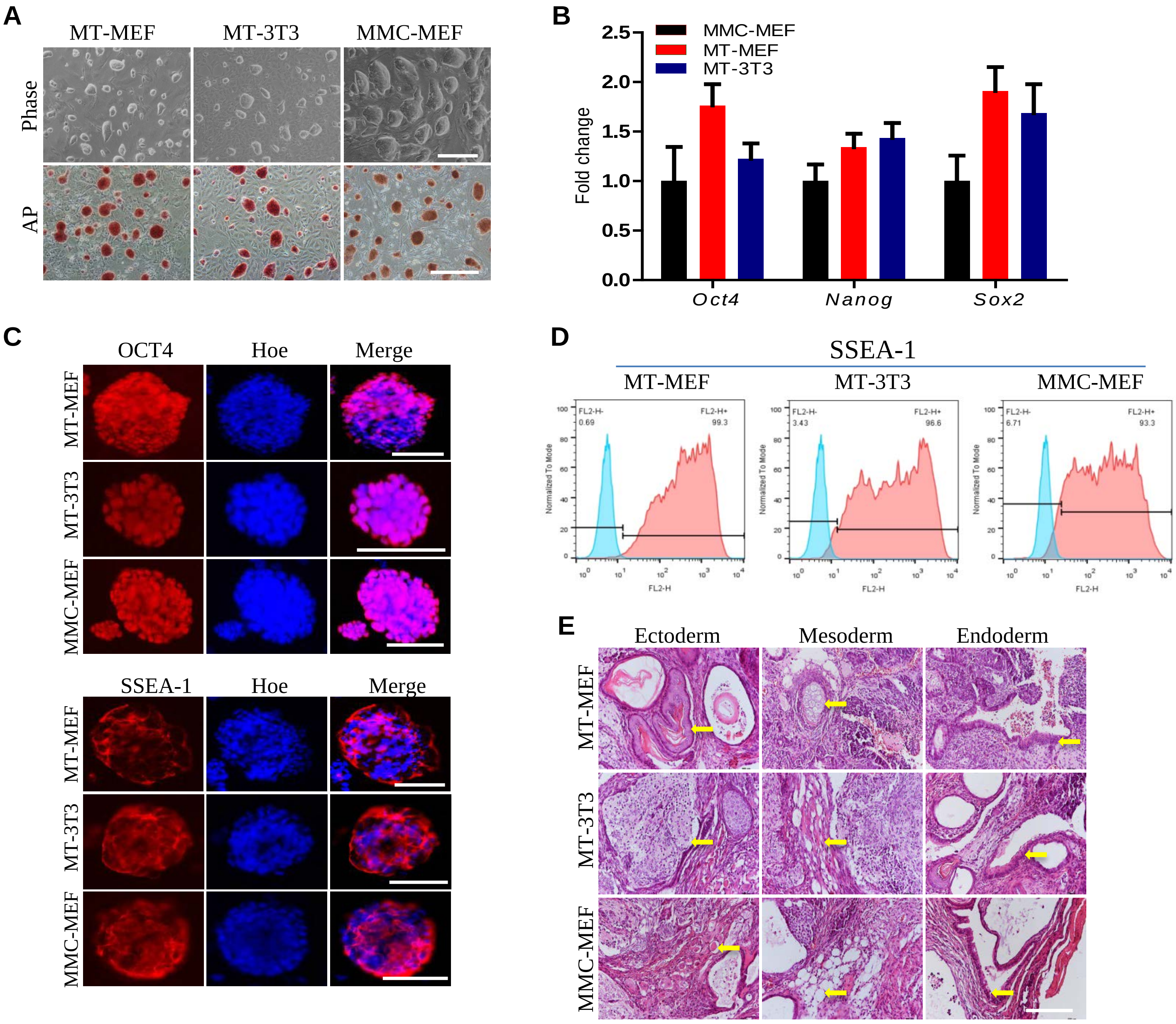

A
MT-MEF
MT-3T3
MMC-MEF
Phase
AP
B
MMC-MEF
MT-MEF
MT-3T3
Fold change
Oct4
Nanog
Sox2
C
OCT4
Hoe
Merge
MT-MEF
MT-3T3
MMC-MEF
SSEA-1
Hoe
Merge
MT-MEF
MT-3T3
MMC-MEF
D
SSEA-1
MT-MEF
MT-3T3
MMC-MEF
E
Ectoderm
Mesoderm
Endoderm
MT-MEF
MT-3T3
MMC-MEF

Figure S7

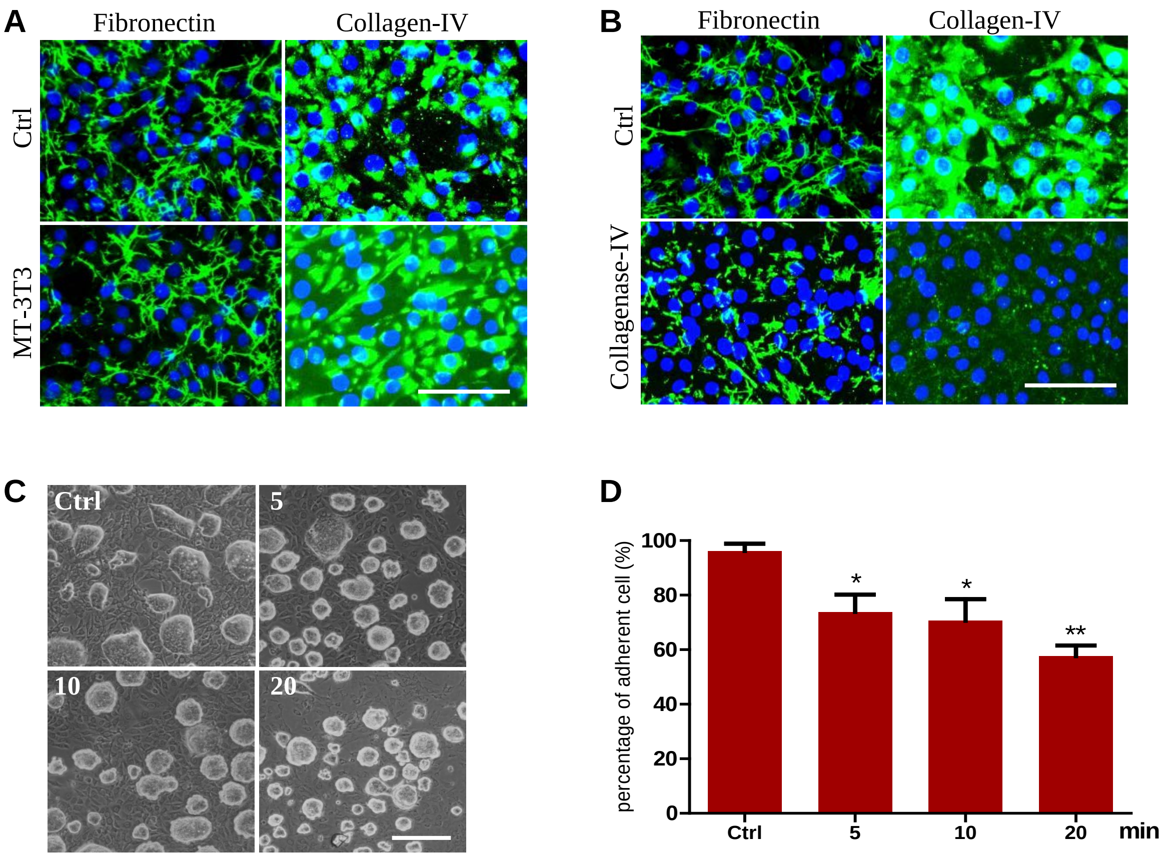
A
Fibronectin
Collagen-IV
Ctrl
MT-3T3
B
Fibronectin
Collagen-IV
Ctrl
Collagenase-IV
C
Ctrl
5
10
20
D
percentage of adherent cell (%)
100
80
60
40
20
0
*
*
**
Ctrl
5
10
20
min

The original gel image of Figure 4-E

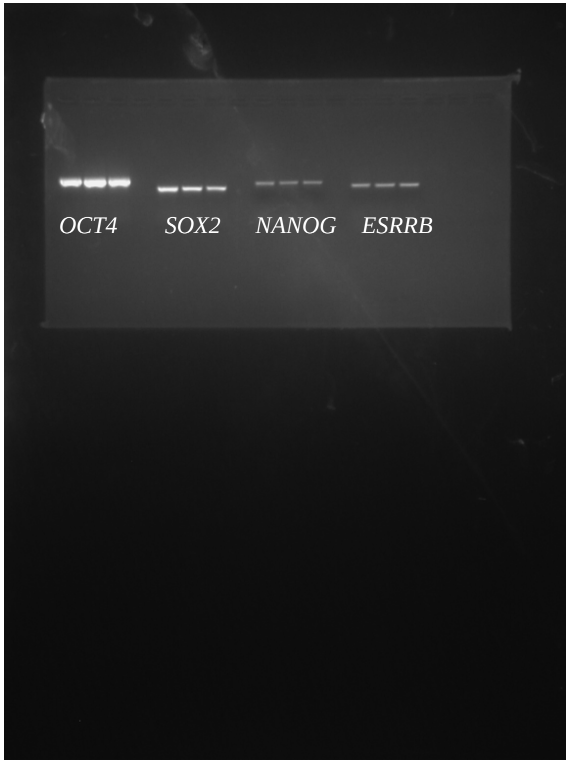

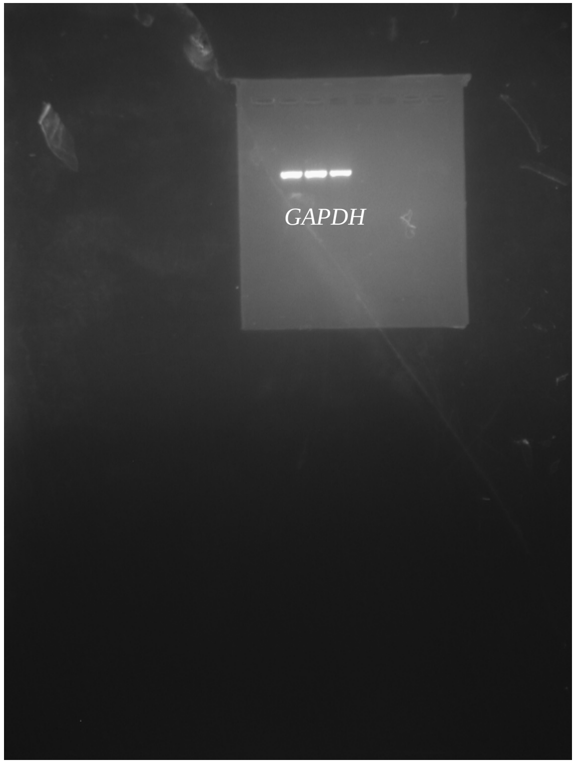

The OCT4, SOX2, NANOG, and ESRRB were cropped from the same gel, GAPDH was cropped from another gel, but this was the same experiment.
